# Supplementary material for: Causal Relationship between Aging and Anorexia Nervosa: A White-Matter-Microstructure-Mediated Mendelian Randomization Analysis
Source: Biomedicines. 2024 Aug 16;12(8):1874. doi: 10.3390/biomedicines12081874 (PMC11351342; doi:10.3390/biomedicines12081874)
Supplement: Supplementary file 1 [file biomedicines-12-01874-s001.zip › Supplementary Figure/Figure S4/Singe SNP plot of MR analysis for TL to ubm-b-2004.pdf]

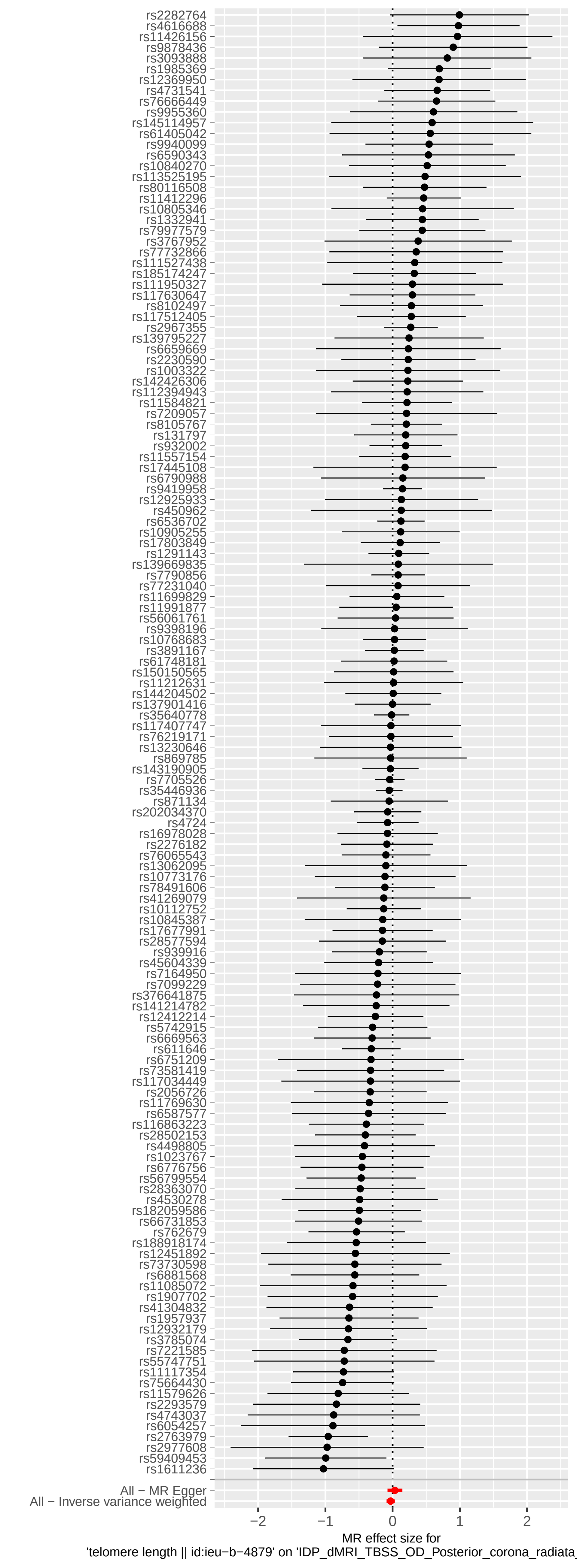
rs2282764
rs4616688
rs11426156
rs9878436
rs3093888
rs1985369
rs12369950
rs4731541
rs76666449
rs9955360
rs145114957
rs61405042
rs9940099
rs6590343
rs10840270
rs113525195
rs80116508
rs11412296
rs10805346
rs1332941
rs79977579
rs3767952
rs77732866
rs111527438
rs185174247
rs111950327
rs117630647
rs8102497
rs117512405
rs2967355
rs139795227
rs6659669
rs2230590
rs1003322
rs142426306
rs112394943
rs11584821
rs7209057
rs8105767
rs131797
rs932002
rs11557154
rs17445108
rs6790988
rs9419958
rs12925933
rs450962
rs6536702
rs10905255
rs17803849
rs1291143
rs139669835
rs7790856
rs77231040
rs11699829
rs11991877
rs56061761
rs9398196
rs10768683
rs3891167
rs61748181
rs150150565
rs11212631
rs144204502
rs137901416
rs35640778
rs117407747
rs76219171
rs13230646
rs869785
rs143190905
rs7705526
rs35446936
rs871134
rs202034370
rs4724
rs16978028
rs2276182
rs76065543
rs13062095
rs10773176
rs78491606
rs41269079
rs10112752
rs10845387
rs17677991
rs28577594
rs939916
rs45604339
rs7164950
rs7099229
rs376641875
rs141214782
rs12412214
rs5742915
rs6669563
rs611646
rs6751209
rs73581419
rs117034449
rs2056726
rs11769630
rs6587577
rs116863223
rs28502153
rs4498805
rs1023767
rs6776756
rs56799554
rs28363070
rs4530278
rs182059586
rs66731853
rs762679
rs188918174
rs12451892
rs73730598
rs6881568
rs11085072
rs1907702
rs41304832
rs1957937
rs12932179
rs3785074
rs7221585
rs55747751
rs11117354
rs75664430
rs11579626
rs2293579
rs4743037
rs6054257
rs2763979
rs2977608
rs59409453
rs1611236
All − MR Egger
All − Inverse variance weighted
−2
−1
0
1
2
MR effect size for
'telomere length || id:ieu−b−4879' on 'IDP_dMRI_TBSS_OD_Posterior_corona_radiata_
